# Role of Individual, Social and Health Factors as Determinants of COVID-19 Vaccine Hesitancy: Results from the Second Phase of the Italian EPICOVID19 Web-Based Survey

**DOI:** 10.3390/ijerph22020314

**Published:** 2025-02-19

**Authors:** Fulvio Adorni, Chiara Cavigli, Nithiya Jesuthasan, Liliana Cori, Aleksandra Sojic, Fabrizio Bianchi, Olivia Curzio, Federica Prinelli

**Affiliations:** 1 Institute of Biomedical Technologies of the National Research Council, ITB-CNR, Segrate, 20090 Milano, Italy; fulvio.adorni@itb.cnr.it (F.A.); nithiya.jesuthasan@itb.cnr.it (N.J.); aleksandra.sojic@itb.cnr.it (A.S.); federica.prinelli@itb.cnr.it (F.P.); 2 Institute of Clinical Physiology of the National Research Council, IFC-CNR, 56124 Pisa, Italy; chiaracavigli@cnr.it (C.C.); liliana.cori@cnr.it (L.C.); fabrizio.bianchi@cnr.it (F.B.)

**Keywords:** COVID-19, vaccine acceptance, SARS-CoV-2, anti-SARS-CoV-2 vaccination, vaccine hesitancy, vaccine refusal, determinants, web-based survey, observational study

## Abstract

Background: Despite scientific breakthroughs in vaccine development, some people remain reluctant to accept the anti-SARS-CoV-2 vaccine. This study evaluates attitudes and behaviours towards the vaccine and factors associated with refusal/hesitancy at the start of Italy’s vaccination campaign. Methods: EPICOVID19 is a two-phase observational web-based study where adult volunteers completed questionnaires in April–June 2020 and January–February 2021. Refusal/hesitancy towards the vaccine was assessed among those not yet vaccinated. We analysed factors associated with refusal/hesitancy by applying multivariate multinomial logistic regression models. Results: Among 36,820 survey participants (mean age of 51 years, 59.7% women, 63.6% highly educated), 2449 (6.7%) were against or hesitant, 4468 (12.1%) were inclined but unsure, and 29,903 (81.2%) were willing to be vaccinated. Factors positively associated with refusal/hesitancy included female sex, middle age, at-risk occupations, medium and low education, deprived status, being underweight, previous SARS-CoV-2 positivity, poor perceived health, no fear of contracting SARS-CoV-2, the fear of contaminated food and natural disasters, and low trust in science, media, government, or institutions. Low hesitancy was associated with student and retired status, overweight and obesity, moderate and high alcohol consumption, no concern about economic and working conditions, and sensitivity to climate change/environmental pollution and epidemics. Conclusions: This survey showed that, during the first month of Italy’s vaccination campaign, some individuals were reluctant to receive the anti-SARS-CoV-2 vaccine. This study highlights potential target groups for tailored communication and prevention campaigns.

## 1. Introduction

Vaccination is the most important medical intervention ever introduced and, together with improved hygiene practises and antibiotics, has eliminated much of the mortality caused by infectious diseases [1]. Since Edward Jenner developed the first formal vaccine against smallpox, in 1796, when he noticed that exposure to cowpox could protect against smallpox [2], many new vaccines have been introduced over the last century.

Nevertheless, since the first vaccine was administered, many people have become hesitant about vaccines, doubting their benefits, and future side effects, worrying about their safety, or questioning the need for them [3]. The most notable episode that spread suspicion about vaccines was the paper published by Wakefield and colleagues in *The Lancet* in 1998, claiming that the measles, mumps, and rubella vaccine caused autism and inflammatory bowel disease in children [4]. Although the journal retracted these findings, the news later reached social media, which has been the incubator of misinformation, disinformation, rumours, and conspiracy theories about vaccination [5].

Vaccine hesitancy (VH) is defined as ’the delay in accepting or refusing vaccination despite the availability of vaccination services’ [6]. The World Health Organization (WHO) Strategic Advisory Group of Experts (SAGE) has identified a wide range of determinants of VH. They can be characterised as contextual (including the historical, social, cultural, environmental, economic, political and institutional factors that may influence vaccine hesitant populations), individual and group influences (personal perceptions or beliefs about vaccines and influences from the social environment), and vaccine or vaccine-specific issues (access, financial cost, the lack of provider recommendation, vaccine novelty, and inconsistent advice from providers) [7].

Numerous reviews and meta-analyses [8,9,10,11] have been published from different parts of the world to investigate the factors associated with COVID-19 VH. The most commonly reported factors are young age, female sex, low education and socioeconomic status, the perceived risk of infection, the presence of comorbidities, poor health status, perceived vulnerability [12], previous vaccine refusal, low trust in scientific research, healthcare systems and governments [12,13,14], the lack of cue to action (i.e., physician recommendation) [12], and the low use of traditional sources of information [15]. In addition to those who refuse vaccination altogether, there is a segment of the population that is sceptical about the Severe Acute Respiratory Syndrome (SARS)-CoV-2 vaccines [16]. Vaccine refusal and hesitancy have become critical barriers to achieving high COVID-19 vaccine coverage [15].

Understanding the factors that contribute to VH is essential for designing appropriate public programmes and communication strategies to inform institutions, promote effective protective behaviours, and implement recommendations to better manage future pandemics. Uncertainties about the safety of the vaccine, probably partly due to communication failures in the various mass media, together with the mistrust of institutions, indicate the importance of addressing this phenomenon and analysing changes over time in the factors associated with this type of perception and behaviour among citizens [17]. Patterns of reluctance need to be monitored in order to develop targeted messages that address the obstacles faced by specific groups [12]. However, a few studies have investigated the attitudes and factors associated with COVID-19 VH or refusal in the Italian general population, taking into account a wide range of sociodemographic, psychological, and behavioural conditions at the beginning of the vaccination campaign [18,19,20,21,22,23].

This study evaluates attitudes and behaviours towards the vaccine and factors associated with refusal/hesitancy at the start of the Italian vaccination campaign. In fact, this study specifically addresses the interaction between risk perception, trust in information, trust and vaccination hesitancy, which have not been adequately addressed in research carried out in Italy. This is relevant because a number of cognitive and emotional factors may influence personal behaviour and allow the identification of different groups of people to be reached by specific interventions. Vaccination hesitation in the context of a pandemic is influenced by the fear of contagion for oneself or one’s family members and by the perception of social or environmental risks in general. This type of sensitivity defines different profiles and clusters. Trust in information, science, and institutions also contributes to defining people’s self-efficacy and ability to act positively in a collective emergency context.

In a previous descriptive manuscript [24], we highlighted the factors prevalent among those who were hesitant or opposed to the anti-SARS-CoV-2 vaccine. We now investigate this phenomenon in more detail, considering all the variables collected in Phase II of the Italian National Epidemiological Survey on COVID-19 (EPICOVID19) [25], paying particular attention to the role of information sources and risk perception.

## 2. Materials and Methods

### 2.1. Study Design and Setting

In April–May 2020, a few weeks after the SARS-CoV-2 outbreak, 198,822 residents of Italy aged 18 years or older voluntarily participated in Phase I of EPICOVID19 [26] by providing online informed consent to participate in this study, which was promoted through numerous forms of media. Approximately half of this self-selected sample consented and agreed to be contacted again for future population surveys on the spread of this emerging virus by providing their personal email address. In November 2020, a second massive wave of infections hit Italy, prompting us to contact participants to complete another online questionnaire. This Phase II of EPICOVID19 was conducted from 15 January to 28 February 2021. During this period, Italy implemented containment measures [27,28] to reduce the spread of infection and the first available vaccines against SARS-CoV-2 were administered to limited groups of the population, such as healthcare workers and people aged 80 or more years starting from December 31st 2020. For the present study, we analysed a total of 41,473 residents from all Italian regions who received an e-mail invitation with a personal link that allowed them to respond to the second questionnaire after completing the informed consent form (response rate 39.4%), including 4653 who had already received the anti-SARS-CoV-2 vaccine (N = 2282 first dose and N = 2371 both doses). The remaining 36,820 participants who had not yet received the vaccine were included in this analysis. A more detailed description of the two study phases can be found elsewhere [25].

### 2.2. Development of the Questionnaire

The Phase II questionnaire was carefully designed following an extensive literature review of existing research to ensure optimal harmonisation and comparability with other large population studies, as previously described [25]. The majority of items were selected on the basis of standardised and validated scales. Prior to release, we conducted a thorough assessment of item clarity to minimise potential misunderstandings and enhance readability. To achieve this goal, we recruited a convenience sample of 20 external volunteers, aged between 18 and 70 years, balanced by gender and representing different educational backgrounds. These volunteers were tasked with completing the questionnaires and providing feedback on their comprehensibility and ease of completion. Based on the feedback received, we refined the questionnaire by adjusting the flow of questions and simplifying the language to ensure clarity and accessibility. Thus, participants were asked to complete a self-administered 75-item questionnaire, which consisted mainly of mandatory and closed questions divided into 7 different sections: (1) sociodemographic data; (2) clinical evaluation; (3) personal characteristics and health status; (4) lifestyles and behaviours; (5) housing conditions; and (6) sources of information and risk perception.

### 2.3. Exposures

The independent variables included in the present study were age, sex, pregnancy status, education, employment and job position at risk of infection, the geographical area of residence, and socioeconomic status, assessed by calculating Townsend Deprivation Scores (TDSs), as previously described in detail [25]. Regarding health status, we considered body mass index (BMI) categorised as healthy weight/underweight/overweight/obese; the number of chronic diseases; smoking habit; alcohol consumption; self-perceived health status; perceived stress; and COVID-19-related variables such as being a COVID-19 case with at least one positive nasopharyngeal swab (NPS) or serological test (ST) result and having received the anti-SARS-CoV-2 vaccine [25].

The fear of infection for oneself or one’s relatives and fear for one’s own and one’s relatives’ economic and work situation were assessed by four questions answered on a Likert scale from 1 to 5 [24]. Participants were asked to indicate their perception of risk for nine topics: crime, terrorism, poverty, unemployment, climate change, environmental pollution, contaminated food, epidemics, and natural disasters. For each of the nine questions, the possible responses were ’low’, ’medium’, ’high’ or ’don’t know’. The questionnaire also asked what sources of information respondents usually consulted and how much trust they had in them. The sources were television, newspapers, radio, government/institutions, media websites, scientists/researchers, science online, associations/trade unions, religious institutions, internet search engines, and social media. It was possible to choose only one of the following four answers for each of the sources regarding consultation and trust: “Yes and trust”, “Yes and partly trust”, “Yes and not trust”, and “Does not consult/does not answer”.

Within the questionnaire used, the key scales for measuring risk perception and trust in information sources were extracted from the Spatial Perception of Risk (PRITASC) survey, developed under the supervision of an international committee [29]. The nine questions concerning PR and the eleven questions concerning trust in information sources were selected. Respondents were asked to express themselves using a four-point Likert-type scale [30].

### 2.4. Main Outcomes

The main outcome measures capture personal intentions to be vaccinated. Participants who had not received the anti-SARS-CoV-2 vaccine were asked to indicate their intentions by selecting one of the following responses: (i) I will get the vaccine, (ii) I will probably get the vaccine but I want to find out more first, (iii) I will probably not get the vaccine but I will get better information anyway, (iv) I will not get the vaccine, and (v) I can’t answer at the moment. The five-level variable was then transformed into a three-level ordinal variable indicating decreasing propensity to vaccinate: “1—definitely yes”, “2—probably yes”, and “3—probably or definitely no”. Vaccine hesitancy refers to the likelihood of choosing vaccination in the future and was investigated through a specific question: the question was simplified from the current literature on hesitancy to choose a significant variable [24].

### 2.5. Statistical Analysis

All variables are expressed as categorical, and results are presented as counts and percentages in rows and columns. The main analysis was performed after excluding individuals who had already been vaccinated against SARS-CoV-2 at the time of answering the questionnaire.

In the analysis of risk perception and trust in information sources, we performed data reduction analysis using a combination of optimal scaling techniques for ordinal variables [31] and Hierarchical Cluster Analysis to highlight similarities in responses and facilitate understanding. We used the Multiple Correspondence Analysis (MCA) method of dimension reduction for multivariate categorical data with the Varimax rotation criterion to facilitate the reading of the association of variables with the MCA components. As for the Hierarchical Cluster Analysis, the results were obtained by choosing the Squared Euclidean Distance as distance measure and the Ward’s minimum variance method for generating clusters. Based on the results of these reduction methods, we were able to group the original variables into a set of new variables with values normalised to the same levels as the original responses.

Multinomial logistic regression models were used to estimate the crude and adjusted odds ratios (ORs and aORs) with 95% confidence intervals (95% CIs) of factors associated with refusal/hesitancy. To assess the impact of potential selection bias due to the exclusion of vaccinated individuals from this analysis, we performed a sensitivity analysis using the same multivariate regression on the entire population of respondents. A further sensitivity analysis was carried out by including in the multivariate model the fragility index [32] calculated at the municipal level based on the place of residence provided by the ISTAT (Italian National Statistics Institute). According to this index, which is based on twelve indicators [33], each Italian municipality is assigned a score from 1 to 10 (1st to 10th decile of the distribution of values), representing increasing levels of fragility. The concept of municipality’s fragility is understood as its exposure to risks of natural and anthropogenic origin and to critical conditions linked to the main demo-social characteristics of the population and the economic-productive system.

The data processing and analysis procedures can therefore be summarised as follows.

Step 1—Among all the exposures considered, a first set of variables was defined that did not undergo transformation and were analysed in subsequent steps 4–6 as directly rated by the participants.

Step 2—A second set of variables was generated, including all those that underwent recoding with direct transformation from the original values and according to the methods previously described in point 2.3.

Step 3—The variables related to risk perception and trust were treated with data reduction techniques that allowed the generation of new variables of a third set.

Step 4—All variables belonging to the three sets were described and then individually analysed in multinomial regression models to infer the crude ORs with respect to the outcomes.

Step 5—Multivariable regression analysis was carried out to obtain independent estimates of the association between all exposures and anti-SARS-CoV-2 vaccine refusal/hesitancy.

Step 6—Using the same statistical models, two sensitivity analyses were performed to evaluate the consistency of the results obtained in the previous step 5.

In all analyses, a value of *p* < 0.05 was considered statistically significant. All statistical analyses were performed using the software packages STATA (version 15, StataCorp LP, 347 College Station, TX, USA) and SPSS (IBM Corp. Released, IBM SPSS Statistics version 25.0, Armonk, NY, USA: IBM Corp.).

## 3. Results

### 3.1. Characteristics of the Respondents

At the time of completing the questionnaire, 4653/41,473 respondents (11.2%) had already been vaccinated. The population in the main analysis therefore consisted of 36,820 respondents who had not received the vaccine. Of these, 2449 (6.7%) were opposed or hesitant, 4468 (12.1%) were inclined but not sure, and 29,903 (81.2%) participants said they were willing to be vaccinated. The majority of the sample was female (59.7%), aged 40 years and older (77.3%), employed in low-risk occupations (48.2%), highly educated (63.6%), had a low deprivation score (60.8%), lived in Northern Italy (70.1%), had a healthy weight (57.4%), no comorbidities (62.2%), were non-smokers (57.2% never-smokers; 81.9% non-smokers), consumed alcohol less than once a week (43.3%, low alcohol consumers), had good perceived health (78%), low-to-moderate perceived stress (92.5%), high or moderate fear of contracting SARS-CoV-2 for themselves and family members, and no fear of economic and working conditions for themselves and family members (Table 1).

### 3.2. Risk Perception and Sources of Information Analysis

**Figure 1:**
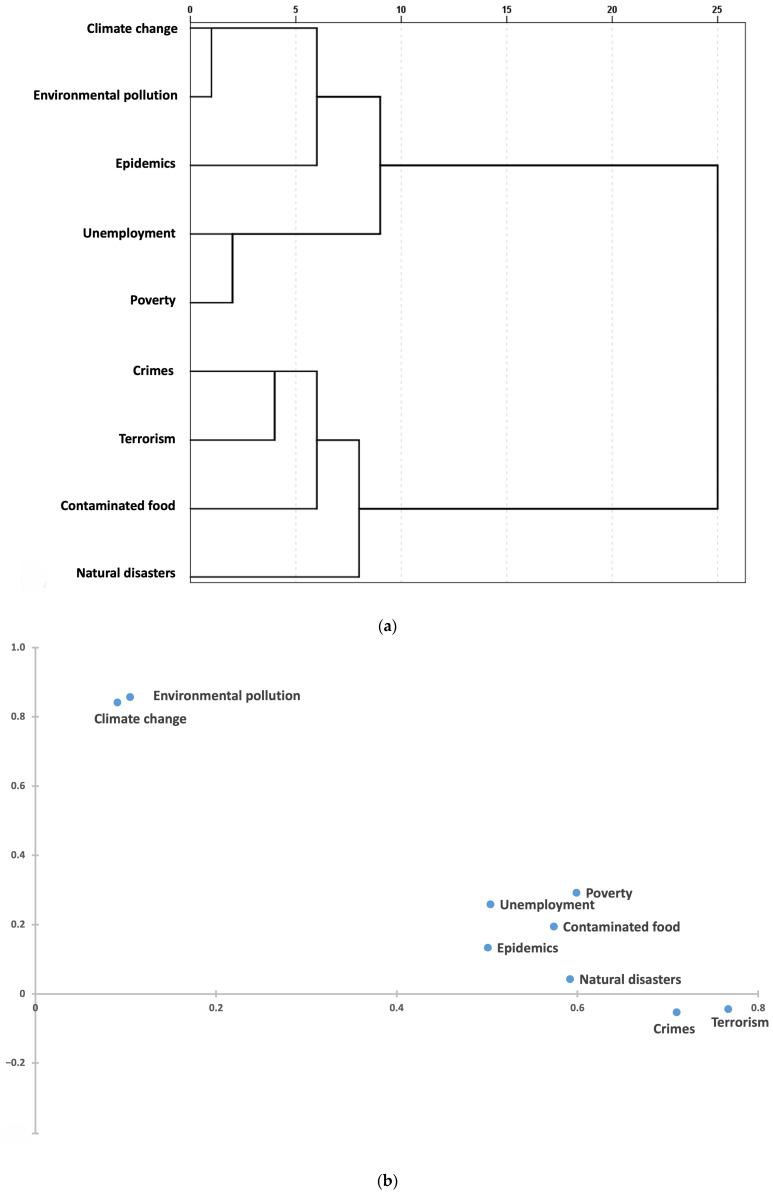
(**a**). Upper figure: Dendrogram of the perceived risk variables (Hierarchical Cluster Analysis). (**b**). Lower figure: Biplot (after Varimax rotation) of the perceived risk variables (Multiple Correspondence Analysis).

**Figure 2:**
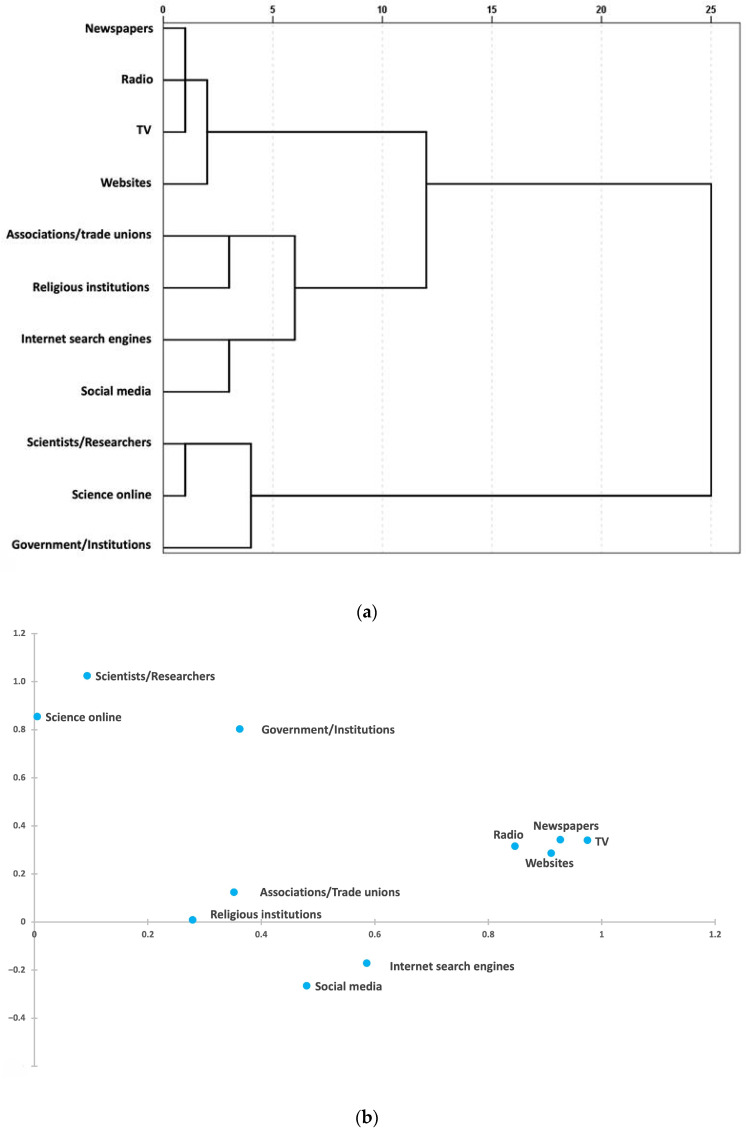
(**a**). Upper figure: Dendrogram of the information sources and trust variables (Hierarchical Cluster Analysis). (**b**). Lower figure: Biplot (after varimax rotation) of the information sources and trust variables (Multiple Correspondence Analysis).

In the analysis of risk perception and sources of information and trust, the data reduction analyses allowed us to define new sets of variables (Figure 1 and Figure 2).

In terms of risk perception, we found two separate components that clustered together: people who were sensitive to the global threat of pollution and climate change, and those who were more concerned about poverty, unemployment, contaminated food, epidemics, natural disasters, crime, and terrorism (Figure 1a,b, Table 2).

Regarding sources of information and trust, five new variables were derived: scientists and researchers/science online, associations and trade unions/religious institutions, internet search engines/social media, TV/radio/newspapers/traditional media websites, and the last one consisting of government and institutions (Figure 2a,b, Table 3).

### 3.3. Factors Associated with Refusal/Hesitancy

In the univariate multinomial logistic regression models, we observed that female sex (both non-pregnant and pregnant), middle age, health workers and other high-risk occupations as well as unemployment, medium and low education, medium and high deprivation scores, underweight and previous SARS-CoV-2 positivity were factors positively and significantly associated with higher hesitancy to anti-SARS-CoV-2 vaccination. Being a student, being retired, being overweight, and reporting moderate and high alcohol consumption were associated with low hesitancy. Poor perceived health status, high perceived stress, and no fear of contracting SARS-CoV-2 for oneself and family members were positively associated with refusal/hesitancy, whereas concern about economic and working conditions for oneself and family members was inversely associated (Supplementary Table S1a).

People who were concerned about natural disasters, poverty or unemployment, crime or terrorism, and contaminated food were less likely to be vaccinated, while those who were concerned about climate change, environmental pollution, and epidemics were more likely to be vaccinated. Regarding the source of information consulted, the majority of respondents said they received information from governments/institutions (88.3%). Those who had low trust in all sources were more reluctant to be vaccinated, particularly mistrusting science, traditional mass media, and government/institutions (Supplementary Table S1b).

**Figure 3:**
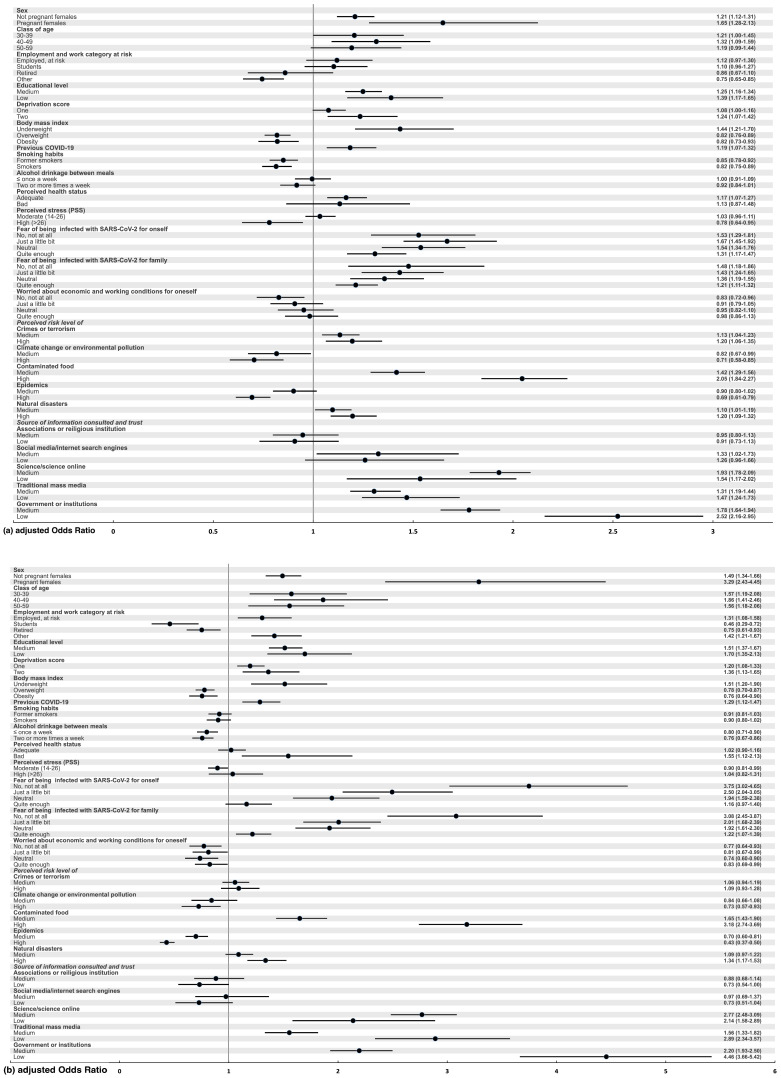
(**a**). Upper figure: Forest plot—propensity to SARS-CoV-2 vaccination and vaccine hesitancy—Multivariable analysis—probably yes; (**b**). Lower figure: Forest plot—propensity to SARS-CoV-2 vaccination and vaccine hesitancy—Multivariable analysis—no or probably no.

The independent association of all variables with the hesitancy or refusal of the anti-SARS-CoV-2 vaccine was assessed by applying a multivariate multinomial logistic regression model (Figure 3a,b and Supplementary Table S2).

We found that higher odds of vaccine hesitancy or vaccine refusal (no or probably not) were observed among females, particularly among pregnant females who had a more than three-fold increased odd of refusal with respect to men. Respondents aged 30–59 years, persons with underweight BMI or with higher deprivation scores or with medium/low educational attainment were positively associated with refusal (aOR ranging from 1.5 to 1.9). A moderate but significant increase in the odds of reluctance was the result for healthcare workers and those in other high-risk occupations, for those who had previously had COVID-19, and for those with poor perceived health status. Instead, significantly lowered odds of vaccine hesitancy/refusal were estimated for students (halved aOR of reluctance), those who were retired (20% reduction in the odd), for those who were overweight or obese, and for those with moderate-to-high alcohol consumption. We also observed that a lower fear of contracting SARS-CoV-2 for oneself or family members was associated with a more than tripled odd of vaccine refusal, while those who were neutral or unconcerned about economic/working conditions were more likely to get vaccinated. People most concerned about climate change/environmental pollution and epidemics were significantly less likely to be vaccine-hesitant (point estimates 0.7 and 0.4, respectively). On the other hand, we found a significant positive association with reluctance for people who were most concerned about natural disasters and, to an even stronger extent, for those concerned about contaminated food. Finally, lower trust in science, traditional mass media and government institutions was strongly associated with higher vaccine hesitancy, with odds increased by 2.1 to 4.5-fold compared to high levels of trust. Conversely, participants who had little trust in information received from associations/religious institutions or social media/internet showed a 30% reduction in the odds of vaccine refusal.

## 4. Discussion

The present study analysed attitudes towards the anti-COVID-19 vaccine and the sociodemographic, clinical, psychological and behavioural factors associated with VH in a large sample of adult volunteers residing in Italy, who participated in Phase II of the web-based EPICOVID19 survey between January and February 2021. This period coincided with initial phases of the vaccination campaign in Italy after the so-called “Vaccine day” of 27 December 2020 that marked the official start of the vaccination campaign against COVID-19 throughout Europe. The results showed important differences in VH according to sex, age, socioeconomic status, the fear of infection, health status and behaviour. This research will inform public health communication on the willingness of unvaccinated people to vaccinate against COVID-19. The results should guide the design of strategies to increase vaccine uptake [34] and educational activities in preparation for vaccination campaigns; this elaboration aimed to create useful communication indications, considering the perplexities of being unvaccinated concerning the vaccination campaign. “Vaccine hesitancy is a state of indecision and uncertainty about vaccination before a decision is made to act (or not to act)” [35]. In the sample of respondents, a small percentage expressed VH: it is this part of the population that is of interest to understand what corrective measures might be taken in information, education, and awareness-raising campaigns to further reduce this percentage [36].

The present study showed that several factors influence the vaccine uptake, as the previous literature from other countries has shown. VH is conceptualised in the literature as involving cognition or affect, behaviour, and decision making. A variety of methods have been used to measure hesitancy [37]. Reasons for hesitancy include the safety and efficacy of vaccination, the belief that vaccination is unnecessary, and a lack of trust in biomedical research [38] and in the health and surveillance system [39,40,41].

One of the most common reasons for hesitancy is a lack of confidence in the COVID-19 vaccine. A low fear or no fear of COVID-19 infection [42], unstable employment status, decreased family income, and worsening health status are predictive factors [43,44].

### 4.1. Attitudes Towards Anti-COVID-19 Vaccine

We found that 81.2% of participants were willing to be vaccinated, while 18.8% were hesitant or opposed to the vaccine. Previous Italian studies performed during the same period reported heterogeneous results. Zarbo [18] and colleagues found that, in a sample of 2015 Italians surveyed between March and May 2021, the proportion of people who accepted the vaccine was 64.6%, those who refused were 6.8%, and those who were doubtful (neither in favour nor against) were 28.5%. In another cross-sectional online survey, conducted in January 2021 on a representative random sample of 1011 citizens from the Emilia-Romagna region, 31.1% of the sample reported hesitancy [23]. In November 2020, Fedele et al. [22] reported vaccine acceptance of just over 26% in a sample of 640 parents from a known paediatric population in Naples. In a nationally representative survey conducted in September 2020 among 1055 Italians aged 15–85 years, 53.7% would accept a potential COVID-19 vaccine (r17). Graffigna et al. [21] surveyed a sample of 1004 middle-aged Italians and found that 58.6% were likely to accept the vaccine in September 2020. On January 22, after one year of the vaccination campaign, in Italy, the actual vaccination coverage rate against COVID-19 was 84%, in line with the results of the present study [45].

### 4.2. Sociodemographic, Clinical, and Lifestyle Factors

VH was more common among women, particularly pregnant women, indicating the need for targeted information campaigns for this group. Among different age groups, hesitancy was highest in the “40–49” age class and lower in “30–39” and “50–59” age groups, and even lower in the “≥60” and “19–29” age groups. Since media and official channels emphasised that people over 60 have an increased risk of complications due to the infection, a low VH in this group was expected. In the same manner, a higher VH might be explained with a less emphasised risk in the under-60 age groups. However, the lower VH among young people does not fit such an explanation. Indeed, several studies reporting a high willingness to vaccinate among young people indicate, as the main motivation for vaccination, altruistic factors such as perceived benefits for others’ health, fear for family members, and benefits for society [46,47,48]. Altruistic motivations for the vaccination could also explain the observed clustering of individuals with environmental concerns as overlapping with the inclination towards vaccination. In addition, younger people have been motivated to vaccinate to travel [48] or to overcome the restrictions related to the pandemic lockdown.

The unemployed were particularly reluctant, while students and retirees were the least reluctant. A specific consideration must be made regarding the results of VH among healthcare workers. At the time of this survey, 71.9% of healthcare workers participating in this study had already been vaccinated and were therefore excluded from the main analyses. This meant that the most hesitant among them were all included in the main analysis, leading to a completely unexpected aOR = 1.4 (95% CI 1.1–1.8). We therefore decided to add already vaccinated healthcare personnel to the category of those definitely willing to be vaccinated in a sensitivity analysis that included all 41,473 respondents. After performing this analysis, the measure of association of health staff with VH was profoundly reversed and settled on an estimated aOR = 0.4 (95% CI 0.3–0.5) of not getting the vaccine. We also tried to estimate the model by including the Municipal Fragility Index provided by the ISTAT as an additional predictor, assuming that it would be able to explain even better the variability observed in the refusal/hesitancy tendency, given its ability to capture the main territorial, environmental and socio-economic dimensions of fragility in the respondents’ municipalities of residence. However, we did not find evidence of a relationship between this composite index and our response, so we decided to exclude it from the final parameterisation.

Low education levels were also associated with high VH. These results indicate the need for vaccination strategies focusing on the specific education of population strata with a lower educational background. Hesitancy increased with higher deprivation levels and was slightly higher among individuals with three or more comorbidities. Those who have previously been infected with COVID-19 were more hesitant, possibly because they consider themselves as already immune [49].

Individuals who perceived themselves as fragile due to physical or psychological conditions tended to be more hesitant. High levels of stress and poor health perception were associated with greater hesitancy, whereas those with better health perceptions showed less hesitancy.

In terms of physical and behavioural characteristics, underweight people were more likely to be hesitant than normal weight people, while overweight and obese people were less reluctant. This might be explained by the fact that media and official information sources emphasised an increased risk of the COVID-19 complications among obese people. Smokers and former smokers were less likely to be hesitant. Interestingly, light and heavy alcohol drinkers were more likely to vaccinate than non-drinkers.

### 4.3. Psychological Factors, Risk Perceived, and Information Sources and Trust

By analysing fear, risk perception, and trust, the results were particularly interesting and suggestive, especially in terms of understanding the factors that influence vaccine acceptance or refusal [50]. Looking at the fear of contracting COVID-19 for oneself, the proportion of those hesitant was higher among those with little or no fear compared to those with high fear and lower among neutrals: compared to the low hesitancy among those who feared being infected, the proportion increased as fear decreased.

When fear for a family member was analysed, the same inverse trend between fear and hesitancy described above was observed: the fear of infection for a family member had a similar pattern to fear for oneself but with a less marked VH increase as fear decreases.

A fear of COVID-19 may play an important role in reducing VH [51]. In the results of the present study, the fear of contagion was one of the psychological states most strongly correlated with vaccine acceptance. As fear increases, VH decreases. The fear of infecting family members led to a greater willingness to vaccinate and thus less hesitancy.

With regard to concern for one’s own economic and employment status, hesitancy was highest among those who stated that they were very worried about work and the economy and decreased as economic/occupational concern decreased: it decreased among those who were sufficiently worried. Hesitancy decreased, as worries about economic and employment status decreased among the most worried, to about half among the neutral and slightly less among the not worried. Concern about the family members’ economic and employment status showed the same pattern of hesitancy as concern about oneself, with slightly less variation. When asked about worrying about the economic and working conditions of family members, hesitancy decreased as worries about themselves decreased.

For all perceived risks, the present results showed that vaccination hesitancy was higher among those who perceived greater risks of unemployment, natural disasters, food contamination, poverty, terrorism, and crime, while—in contrast—hesitancy was lower among those perceiving greater risks of environmental pollution, epidemics, and climate change. Hesitancy decreased in association with an increasing perception of the risks of environmental pollution and climate change. These are the aspects of environmental and health crises that can be considered most closely linked to current events, on which information is constantly available, which can be linked to awareness and—on the other hand—also to trust: those who are most concerned about these issues trust the sources of information and are inclined to vaccinate. Several studies focused the analysis on eco-anxiety and, in particular, the risks to wellbeing and mental health associated with climate change [52] and with specific reference to younger generations [53]. To illustrate eco-anxiety, several studies also mention environmental pollution, bringing together concerns into a complex psychological state of worry that, while unpleasant, could be a powerful motivator. In the case of the present research, we observed that worried people were more inclined to vaccination, which has proved to be a way of overcoming COVID-19, a way of facing the problem and acting to limit it.

More hesitant people could be included in the frail population, with particular attention paid to informing and involving them on an ongoing basis. In general, the results of the present study indicated that VH was associated with a whole range of social, economic, cultural, and even health-related characteristics of frailty.

For all the sources of information considered (television, radio, newspapers, media websites), the hesitation rate compared to the answer “Yes and I trust” increased as trust decreased; in particular, the hesitation rate reaches high values among those who declared that they do not trust TV, radio, newspapers, government/institutions, scientist/researchers, media websites, with the sole exception of social media. The differences were more nuanced for media that are considered as chosen or controlled by the respondent, such as social media and internet search engines. In terms of sources of information, those who use and trust television were more likely to vaccinate, while those who are more mistrustful were also more hesitant about the vaccine. When it comes to information from the internet, trust is less important because the choice of what to consult seems to be personal.

The results obtained here were confirmed by other evidence [54], where the authors find that trust is a key determinant of VH, and, in particular, that trust in social media and its use as a main source of information was positively associated with hesitancy. On the other hand, concern about economic and working conditions, whether for oneself or one’s family, leads to greater hesitancy. This concern could be linked to a social condition in which there is no trust in state institutions and national institutional leadership. Concerns about material conditions thus work oppositely to the fear of contracting COVID-19: a fear that seems to be presumably more irrational. In summary, the reported reasons for hesitancy appeared to be linked to a complex network of interrelated factors, including ’uncertainty’ about the results and efficacy of the vaccine. The ’fear’ of vaccines is driven by multiple rumours and conspiracy theories within the community, lack of control over the behaviour of others, and a desire not to be controlled, especially by the government. At the same time, however, there is a resignation to compulsory vaccination, accompanied by a lack of trust in government, both in relation to vaccines and health messages [55].

Even with compulsory vaccination, reluctance to use COVID-19 vaccines remains a concern among a certain segment of the population with various societal, cultural and health-related frailties. The rapid change in the way that care is experienced today shows that we are moving from a collective to an individual perspective. Social media and smartphones have contributed to this trend, moving people away from face-to-face interactions. In contrast, group identity, belonging, and online support connect individuals, but they can also create divisive and polarised opinions. This may block deeper emotional connections and understanding, leading to the further breakdown of trust and relationships. Polarised opinions may perhaps still create relatively deep emotional connections (based on common emotions of hatred and distrust), although they mostly limit the interactions of digital media participants. The problem may be a closed view and resistance to the argumentation and acceptance of facts from different perspectives. Encouraging the sharing of personal COVID-19 vaccine stories on social media, along with addressing specific vaccine hesitancies and emphasising freedom from fear after vaccination, could help reduce VH in the population [56]. From a public health perspective, it is important to recognise the growing challenges associated with vaccine coverage.

### 4.4. Strengths and Limitations

This study has several limitations. First, the cross-sectional nature of this study limits causal inference; the study design does not allow us to make any assumptions about causality but only to identify possible associations between different characteristics and levels of perplexity on vaccination. Second, the web survey and voluntary participation may be affected by selection bias and limited generalisability to other populations. For example, the enrolled sample was female, younger, healthier, and wealthier than the general population. In addition, the data were self-reported, which may introduce measurement and recall bias. Other weaknesses concern the occurrence of pathologies or conditions that have led to contraindications to the administration of the vaccine, personal changes in the willingness to join the vaccination campaign, and possible delays in administration or the lack of administration due to contracting COVID-19 during the period between the two doses. The present study also has several strengths, including its community-based design, large sample size, and comprehensive data collection on socio-demographic, clinical, behavioural, and psychological factors, mainly using validated scales.

## 5. Conclusions

The factors influencing vaccine acceptance, hesitancy or refusal are complex, but our findings shed some light on socio-demographic, clinical and behavioural determinants. Gender, age, socioeconomic status, body weight, previous SARS-CoV-2 infection, self-perceived health status, fear and trust in the source of information are dominant factors influencing refusal or hesitancy that should be targeted when implementing vaccination campaigns.

Simply providing more information about risks and benefits of vaccines is often insufficient. Effective communication strategies must be targeted and take risk perceptions into account as a constitutive fact [30]. In this respect, it may indeed be possible to provide responses to people’s fear that increase their awareness of their own role in positively changing the situation. This may be particularly true where people feel anxious about environmental problems, which may lead to increased participation in vaccination campaigns and proactive engagement by individuals to involve others.

Then, there are certain people defined as ‘information multipliers’, those who are closest to the patient, and a source of reliable information, namely, doctors and, in particular, general practitioners. Information needs to be articulated in a way that is both simplified and comprehensible and that provides insights and first-hand accounts that help to build and maintain trust. Public health policy, especially concerning COVID-19, requires accurate information and continued trust in institutions and communicators. Campaigns should focus on specific issues, explain the scientific method, facilitate informed choices, and raise awareness through transparent and updated information. In Italy, in particular, there is a need to improve public health literacy, as highlighted by various studies [57].

The WHO has identified essential trust determinants in communication: competence, objectivity, fairness, consistency, sincerity, and trust, which should be integrated into public education [58,59,60]. Innovative public health communication strategies should include and build on these radically relevant factors as determinants of trust and be able to evaluate them for continuous corrections and improvement [61,62]. Evaluation is one of the most critical issues in this area, and the recent experiences of promoting COVID-19 vaccination provide much expertise and lessons for moving forward.

Communication strategies need to address individuals’ perceptions of risk, simplifying information without compromising its accuracy. Personal narratives can humanise data and increase trust. Campaigns should not only describe vaccines but also explain the science behind their safety. Transparency and updates are crucial for informed decision making. In countries like Italy, there is an urgent need to improve health literacy to avoid fear and confusion, through face-to-face interviews, focus groups, social media campaigns and bottom-up citizen-led activities, including participatory epidemiology methods. The WHO emphasises the need for competence, objectivity, consistency and sincerity in communication. Communication strategies must be based on these principles and include continuous feedback to improve them over time, learning from COVID-19 vaccination efforts. Building trust is an ongoing process that requires transparency, empathy, and constant engagement.

## Figures and Tables

**Table 1:** Propensity to/Attitude towards COVID-19 vaccination by demographic, socio-economic, geographical area, clinical characteristics, lifestyles, and self-reported psychological factors.

		Total	Yes	Probably Yes	No or Probably Not
		N (Col%)	N (Row%)	N (Row%)	N (Row%)
		36,820 (100)	29,903 (81.2)	4468 (12.1)	2449 (6.7)
Sex at birth	Males	14,853 (40.3)	12,518 (84.3)	1554 (10.5)	781 (5.3)
	Females	21,967 (59.7)	17,385 (79.1)	2914 (13.3)	1668 (7.6)
	Not pregnant	21,412 (58.2)	16,986 (79.3)	2828 (13.2)	1598 (7.5)
	Pregnant	555 (1.5)	399 (71.9)	86 (15.5)	70 (12.6)
Class of age	19–29	2298 (6.2)	1961 (85.3)	247 (10.7)	90 (3.9)
	30–39	6096 (16.6)	4958 (81.3)	741 (12.2)	397 (6.5)
	40–49	7980 (21.7)	6202 (77.7)	1106 (13.9)	672 (8.4)
	50–59	9521 (25.9)	7377 (77.5)	1322 (13.9)	822 (8.6)
	60+	10,925 (29.7)	9405 (86.1)	1052 (9.6)	468 (4.3)
Employment and work category at risk	Employed, not at risk	17,759 (48.2)	14,413 (81.2)	2190 (12.3)	1156 (6.5)
	Employed, school staff	3319 (9.0)	2695 (81.2)	424 (12.8)	200 (6.0)
	Employed, health staff	989 (2.7)	737 (74.5)	132 (13.3)	120 (12.1)
	Employed, other at risk	1728 (4.7)	1287 (74.5)	263 (15.2)	178 (10.3)
	Unemployed	2584 (7.0)	1901 (73.6)	424 (16.4)	259 (10.0)
	Students	1225 (3.3)	1079 (88.1)	119 (9.7)	27 (2.2)
	Retired	6546 (17.8)	5770 (88.1)	543 (8.3)	233 (3.6)
	Other	2670 (7.3)	2021 (75.7)	373 (14.0)	276 (10.3)
Educational level	High	23,403 (63.6)	19,519 (83.4)	2587 (11.1)	1297 (5.5)
	Medium	12,114 (32.9)	9401 (77.6)	1684 (13.9)	1029 (8.5)
	Low	1303 (3.5)	983 (75.4)	197 (15.1)	123 (9.4)
Deprivation score	0	22,398 (60.8)	18,509 (82.6)	2552 (11.4)	1337 (6.0)
	1	11,763 (31.9)	9394 (79.9)	1505 (12.8)	864 (7.3)
	2	2409 (6.5)	1824 (75.7)	365 (15.2)	220 (9.1)
	3+	250 (0.7)	176 (70.4)	46 (18.4)	28 (11.2)
Geographical area	Northern	25,816 (70.1)	20,883 (80.9)	3169 (12.3)	1764 (6.8)
	Central	7322 (19.9)	5967 (81.5)	894 (12.2)	461 (6.3)
	Southern and islands	3660 (9.9)	3036 (83.0)	402 (11.0)	222 (6.1)
	Unknown	22 (0.1)	17 (77.3)	3 (13.6)	2 (9.1)
BMI	Healthy weight	21,130 (57.4)	16,978 (80.4)	2673 (12.7)	1479 (7.0)
	Underweight	1148 (3.1)	841 (73.3)	191 (16.6)	116 (10.1)
	Overweight	10,792 (29.3)	9016 (83.5)	1165 (10.8)	611 (5.7)
	Obesity	3258 (8.8)	2663 (81.7)	386 (11.8)	209 (6.4)
	Unknown	492 (1.3)	405 (82.3)	53 (10.8)	34 (6.9)
N° of comorbidities	None	22,903 (62.2)	18,548 (81.0)	2806 (12.3)	1549 (6.8)
	One	8577 (23.3)	7010 (81.7)	1027 (12.0)	540 (6.3)
	Two	3466 (9.4)	2844 (82.1)	405 (11.7)	217 (6.3)
	Three or more	1874 (5.1)	1501 (80.1)	230 (12.3)	143 (7.6)
Previous COVID-19	Yes	3753 (10.2)	2844 (75.8)	541 (14.4)	368 (9.8)
Smoker	No	21,057 (57.2)	16,938 (80.4)	2697 (12.8)	1422 (6.8)
	Former smoker	9111 (24.7)	7581 (83.2)	982 (10.8)	548 (6.0)
	Smoker	6652 (18.1)	5384 (80.9)	789 (11.9)	479 (7.2)
Alcohol drinkage between meals	Never	6916 (18.8)	5419 (78.4)	902 (13.0)	595 (8.6)
	≤once a week	15,952 (43.3)	12,900 (80.9)	2023 (12.7)	1029 (6.5)
	Two or more times a week	13,952 (37.9)	11,584 (83.0)	1543 (11.1)	825 (5.9)
Perceived health status	Good	28,731 (78.0)	23,438 (81.6)	3376 (11.8)	1917 (6.7)
	Adequate	7491 (20.3)	6001 (80.1)	1019 (13.6)	471 (6.3)
	Bad	598 (1.6)	464 (77.6)	73 (12.2)	61 (10.2)
Perceived stress (PSS)	Low (<14)	18,069 (49.1)	14,867 (82.3)	1998 (11.1)	1204 (6.7)
	Moderate (14–26)	15,974 (43.4)	12,788 (80.1)	2149 (13.5)	1037 (6.5)
	High (>26)	1368 (3.7)	1100 (80.4)	147 (10.7)	121 (8.8)
	NA	1409 (3.8)	1148 (81.5)	174 (12.3)	87 (6.2)
Fear of being infected with SARS-CoV-2					
For oneself	Yes, a lot	6733 (18.3)	5899 (87.6)	603 (9.0)	231 (3.4)
	Quite enough	12,987 (35.3)	11,003 (84.7)	1518 (11.7)	466 (3.6)
	Neutral	6907 (18.8)	5463 (79.1)	959 (13.9)	485 (7.0)
	Just a little bit	6846 (18.6)	5264 (76.9)	962 (14.1)	620 (9.1)
	No, not at all	3347 (9.1)	2274 (67.9)	426 (12.7)	647 (19.3)
For family members	Yes, a lot	15,346 (41.7)	13,080 (85.2)	1617 (10.5)	649 (4.2)
	Quite enough	13,308 (36.1)	10,862 (81.6)	1729 (13.0)	717 (5.4)
	Neutral	3492 (9.5)	2664 (76.3)	490 (14.0)	338 (9.7)
	Just a little bit	3538 (9.6)	2583 (73.0)	495 (14.0)	460 (13.0)
	No, not at all	1136 (3.1)	714 (62.9)	137 (12.1)	285 (25.1)
Worried about economic and working conditions					
For oneself	Yes, a lot	3963 (10.8)	2933 (74.0)	606 (15.3)	424 (10.7)
	Quite enough	5713 (15.5)	4438 (77.7)	833 (14.6)	442 (7.7)
	Neutral	5857 (15.9)	4758 (81.2)	786 (13.4)	313 (5.3)
	Just a little bit	7179 (19.5)	5886 (82.0)	868 (12.1)	425 (5.9)
	No, not at all	14,108 (38.3)	11,888 (84.3)	1375 (9.7)	845 (6.0)
For family members	Yes, a lot	5054 (13.7)	3890 (77.0)	730 (14.4)	434 (8.6)
	Quite enough	7794 (21.2)	6197 (79.5)	1038 (13.3)	559 (7.2)
	Neutral	5219 (14.2)	4225 (81.0)	704 (13.5)	290 (5.6)
	Just a little bit	7467 (20.3)	6170 (82.6)	874 (11.7)	423 (5.7)
	No, not at all	11,286 (30.7)	9421 (83.5)	1122 (9.9)	743 (6.6)

**Table 2:** Propensity to/Attitude towards COVID-19 vaccination by perceived risks.

		Total	Yes	Probably Yes	No or Probably Not
		N (Col%)	N (Row%)	N (Row%)	N (Row%)
		36,820 (100)	29,903 (81.2)	4468 (12.1)	2449 (6.7)
Perceived risk level of					
Crimes or terrorism	Low	13,579 (36.9)	11,562 (85.1)	1305 (9.6)	712 (5.2)
	Medium	16,218 (44.0)	12,962 (79.9)	2120 (13.1)	1136 (7.0)
	High	4895 (13.3)	3666 (74.9)	776 (15.9)	453 (9.3)
	Doesn’t know/doesn’t answer	2128 (5.8)	1713 (80.5)	267 (12.5)	148 (7.0)
Poverty or unemployment	Low	5198 (14.1)	4431 (85.2)	498 (9.6)	269 (5.2)
	Medium	14,068 (38.2)	11,563 (82.2)	1649 (11.7)	856 (6.1)
	High	15,474 (42.0)	12,219 (79.0)	2067 (13.4)	1188 (7.7)
	Doesn’t know/doesn’t answer	2080 (5.6)	1690 (81.3)	254 (12.2)	136 (6.5)
Climate change or environmental pollution	Low	1125 (3.1)	853 (75.8)	160 (14.2)	112 (10.0)
	Medium	8850 (24.0)	7040 (79.5)	1159 (13.1)	651 (7.4)
	High	24,886 (67.6)	20,424 (82.1)	2907 (11.7)	1555 (6.2)
	Doesn’t know/doesn’t answer	1959 (5.3)	1586 (81.0)	242 (12.4)	131 (6.7)
Contaminated food	Low	9644 (26.2)	8460 (87.7)	805 (8.3)	379 (3.9)
	Medium	13,762 (37.4)	11,342 (82.4)	1630 (11.8)	790 (5.7)
	High	10,505 (28.5)	7744 (73.7)	1677 (16.0)	1084 (10.3)
	Doesn’t know/doesn’t answer	2909 (7.9)	2357 (81.0)	356 (12.2)	196 (6.7)
Epidemics	Low	3626 (9.8)	2764 (76.2)	447 (12.3)	415 (11.4)
	Medium	11,487 (31.2)	9127 (79.5)	1484 (12.9)	876 (7.6)
	High	19,267 (52.3)	16,074 (83.4)	2213 (11.5)	980 (5.1)
	Doesn’t know/doesn’t answer	2440 (6.6)	1938 (79.4)	324 (13.3)	178 (7.3)
Natural disasters	Low	15,454 (42.0)	12,754 (82.5)	1715 (11.1)	985 (6.4)
	Medium	10,828 (29.4)	8757 (80.9)	1370 (12.7)	701 (6.5)
	High	8035 (21.8)	6383 (79.4)	1071 (13.3)	581 (7.2)
	Doesn’t know/doesn’t answer	2503 (6.8)	2009 (80.3)	312 (12.5)	182 (7.3)

**Table 3:** Propensity to/Attitude towards COVID-19 vaccination by source of information consulted and trust.

		Total	Yes	Probably Yes	No or Probably Not
		N (Col%)	N (Row%)	N (Row%)	N (Row%)
		36,820 (100)	29,903 (81.2)	4468 (12.1)	2449 (6.7)
Source of information consulted and trust					
Associations or religious institution	High	2167 (5.9)	1885 (87.0)	194 (9.0)	88 (4.1)
	Medium	7007 (19.0)	5624 (80.3)	908 (13.0)	475 (6.8)
	Low	1737 (4.7)	1318 (75.9)	252 (14.5)	167 (9.6)
	Did not consult/answer	25,909 (70.4)	21,076 (81.3)	3114 (12.0)	1719 (6.6)
Social media/internet search engines	High	942 (2.6)	831 (88.2)	66 (7.0)	45 (4.8)
	Medium	18,737 (50.9)	14,979 (79.9)	2423 (12.9)	1335 (7.1)
	Low	6941 (18.9)	5604 (80.7)	893 (12.9)	444 (6.4)
	Did not consult/answer	10,200 (27.7)	8489 (83.2)	1086 (10.6)	625 (6.1)
Science/science online	High	23,510 (63.9)	20,592 (87.6)	2095 (8.9)	823 (3.5)
	Medium	8901 (24.2)	6003 (67.4)	1704 (19.1)	1194 (13.4)
	Low	458 (1.2)	280 (61.1)	83 (18.1)	95 (20.7)
	Did not consult/answer	3951 (10.7)	3028 (76.6)	586 (14.8)	337 (8.5)
Traditional mass media	High	9338 (25.4)	8430 (90.3)	672 (7.2)	236 (2.5)
	Medium	23,827 (64.7)	18,965 (79.6)	3238 (13.6)	1624 (6.8)
	Low	2069 (5.6)	1283 (62.0)	359 (17.4)	427 (20.6)
	Did not consult/answer	1586 (4.3)	1225 (77.2)	199 (12.5)	162 (10.2)
Government or institutions	High	16,706 (45.4)	15,025 (89.9)	1257 (7.5)	424 (2.5)
	Medium	13,949 (37.9)	10,477 (75.1)	2260 (16.2)	1212 (8.7)
	Low	1838 (5.0)	1053 (57.3)	377 (20.5)	408 (22.2)
	Did not consult/answer	4327 (11.8)	3348 (77.4)	574 (13.3)	405 (9.4)

## Data Availability

The original contributions presented in this study are included in this article and in the Supplementary Material. Further inquiries can be directed to the corresponding author/s. Aggregated data and the analysis source code will be made available upon reasonable request to the corresponding author.

## Supplementary Materials

The following supporting information can be downloaded at https://www.mdpi.com/article/10.3390/ijerph22020314/s1, Supplementary Table S1a: Propensity to SARS-CoV-2 vaccination and vaccine hesitancy—Univariate analysis; and Supplementary Table S1b: Propensity to SARS-CoV-2 vaccination and vaccine hesitancy—Univariate analysis.
