# Supplementary material for: Role of Individual, Social and Health Factors as Determinants of COVID-19 Vaccine Hesitancy: Results from the Second Phase of the Italian EPICOVID19 Web-Based Survey
Source: Int J Environ Res Public Health. 2025 Feb 19;22(2):314. doi: 10.3390/ijerph22020314 (PMC11855322; doi:10.3390/ijerph22020314)
Supplement: Supplementary file 1 [file ijerph-22-00314-s001.zip › ijerph-3317430-supplementary.pdf]

Supplementary Table S1a: Propensity to SARS-CoV-2 vaccination and vaccine hesitancy – Univariate analysis

		Probably yes	No or probably not
		**OR (95% CI)**	**OR (95% CI)**
Sex at birth	Males	1	1
	Females	1,4 (1,3-1,4)	1,5 (1,4-1,7)
	Not pregnant	1,3 (1,3-1,4)	1,5 (1,4-1,6)
	Pregnant	1,7 (1,4-2,2)	2,8 (2,2-3,7)
Class of age	19-29	1	1
	30-39	1,2 (1,0-1,4)	1,7 (1,4-2,2)
	40-49	1,4 (1,2-1,6)	2,4 (1,9-3,0)
	50-59	1,4 (1,2-1,6)	2,4 (1,9-3,0)
	60+	0,9 (0,8-1,0)	1,1 (0,9-1,4)
Employment and work category at risk	Employed, not at risk	1	1
	Employed, school staff	1,0 (0,9-1,2)	0,9 (0,8-1,1)
	Employed, health staff	1,2 (1,0-1,4)	2,0 (1,7-2,5)
	Employed, other at risk	1,3 (1,2-1,5)	1,7 (1,5-2,0)
	Unemployed	1,5 (1,3-1,6)	1,7 (1,5-2,0)
	Students	0,7 (0,6-0,9)	0,3 (0,2-0,5)
	Retired	0,6 (0,6-0,7)	0,5 (0,4-0,6)
	Other	1,2 (1,1-1,4)	1,7 (1,5-2,0)
Educational level	High	1	1
	Medium	1,4 (1,3-1,4)	1,6 (1,5-1,8)
	Low	1,5 (1,3-1,8)	1,9 (1,5-2,3)
Deprivation score	0	1	1
	1	1,2 (1,1-1,2)	1,3 (1,2-1,4)
	2	1,5 (1,3-1,6)	1,7 (1,4-1,9)
	3+	1,9 (1,4-2,6)	2,2 (1,5-3,3)
BMI	Healthy weight	1	1
	Underweight	1,4 (1,2-1,7)	1,6 (1,3-1,9)
	Overweight	0,8 (0,8-0,9)	0,8 (0,7-0,9)
	Obesity	0,9 (0,8-1,0)	0,9 (0,8-1,0)
	Unknown	0,8 (0,6-1,1)	1,0 (0,7-1,4)
N° of comorbidities	None	1	1
	One	1,0 (0,9-1,0)	0,9 (0,8-1,0)
	Two	0,9 (0,8-1,1)	0,9 (0,8-1,1)
	Three or more	1,0 (0,9-1,2)	1,1 (1,0-1,4)
Previous COVID-19	Yes	1,3 (1,2-1,4)	1,7 (1,5-1,9)
Smoker	No	1	1
	Former smoker	0,8 (0,8-0,9)	0,9 (0,8-1,0)
	Smoker	0,9 (0,8-1,0)	1,1 (1,0-1,2)
Alcohol drinkage between meals	Never	1	1
	≤ once a week	0,9 (0,9-1,0)	0,7 (0,7-0,8)
	Two or more times a week	0,8 (0,7-0,9)	0,6 (0,6-0,7)
Geographical area	Northern	1	1
	Central	1,0 (0,9-1,1)	0,9 (0,8-1,0)
	Southern and islands	0,9 (0,8-1,0)	0,9 (0,7-1,0)

	Unknown	1,2 (0,3-4,1)	1,5 (0,3-6,5)
Perceived health status	Good	1	1
	Adequate	1,2 (1,1-1,3)	1,0 (0,9-1,1)
	Bad	1,1 (0,9-1,4)	1,6 (1,2-2,1)
Perceived stress (PSS)	Low (<14)	1	1
	Moderate (14-26)	1,3 (1,2-1,3)	1,0 (0,9-1,1)
	High (>26)	1,0 (0,8-1,2)	1,4 (1,1-1,7)
	NA	1,1 (1,0-1,3)	0,9 (0,7-1,2)
Fear of being infected with SARS-CoV-2			
For oneself	Yes, a lot	1	1
	Quite enough	1,4 (1,2-1,5)	1,1 (0,9-1,3)
	Neutral	1,7 (1,5-1,9)	2,3 (1,9-2,7)
	Just a little bit	1,8 (1,6-2,0)	3,0 (2,6-3,5)
	No, not at all	1,8 (1,6-2,1)	7,3 (6,2-8,5)
For family members	Yes, a lot	1	1
	Quite enough	1,3 (1,2-1,4)	1,3 (1,2-1,5)
	Neutral	1,5 (1,3-1,7)	2,6 (2,2-2,9)
	Just a little bit	1,6 (1,4-1,7)	3,6 (3,2-4,1)
	No, not at all	1,6 (1,3-1,9)	8,0 (6,9-9,4)
Worried about economic and working conditions			
For oneself	Yes, a lot	1	1
	Quite enough	0,9 (0,8-1,0)	0,7 (0,6-0,8)
	Neutral	0,8 (0,7-0,9)	0,5 (0,4-0,5)
	Just a little bit	0,7 (0,6-0,8)	0,5 (0,4-0,6)
	No, not at all	0,6 (0,5-0,6)	0,5 (0,4-0,6)
For family members	Yes, a lot	1	1
	Quite enough	0,9 (0,8-1,0)	0,8 (0,7-0,9)
	Neutral	0,9 (0,8-1,0)	0,6 (0,5-0,7)
	Just a little bit	0,8 (0,7-0,8)	0,6 (0,5-0,7)
	No, not at all	0,6 (0,6-0,7)	0,7 (0,6-0,8)

Supplementary Table S1b: Propensity to SARS-CoV-2 vaccination and vaccine hesitancy – Univariate analysis

Perceived risk level of			
Crimes or terrorism	Low	1	1
	Medium	1,4 (1,3-1,6)	1,4 (1,3-1,6)
	High	1,9 (1,7-2,1)	2,0 (1,8-2,3)
	Doesn't know/doesn't answer	1,4 (1,2-1,6)	1,4 (1,2-1,7)
Poverty or unemployment	Low	1	1
	Medium	1,3 (1,1-1,4)	1,2 (1,1-1,4)
	High	1,5 (1,4-1,7)	1,6 (1,4-1,8)
	Doesn't know/doesn't answer	1,3 (1,1-1,6)	1,3 (1,1-1,6)
Climate change or environmental pollution	Low	1	1
	Medium	0,9 (0,7-1,1)	0,7 (0,6-0,9)
	High	0,8 (0,6-0,9)	0,6 (0,5-0,7)
	Doesn't know/doesn't answer	0,8 (0,7-1,0)	0,6 (0,5-0,8)
Contaminated food	Low	1	1
	Medium	1,5 (1,4-1,7)	1,6 (1,4-1,8)
	High	2,3 (2,1-2,5)	3,1 (2,8-3,5)
	Doesn't know/doesn't answer	1,6 (1,4-1,8)	1,9 (1,6-2,2)
Epidemics	Low	1	1
	Medium	1,0 (0,9-1,1)	0,6 (0,6-0,7)
	High	0,9 (0,8-1,0)	0,4 (0,4-0,5)
	Doesn't know/doesn't answer	1,0 (0,9-1,2)	0,6 (0,5-0,7)
Natural disasters	Low	1	1
	Medium	1,2 (1,1-1,3)	1,0 (0,9-1,1)
	High	1,2 (1,2-1,4)	1,2 (1,1-1,3)
	Doesn't know/doesn't answer	1,2 (1,0-1,3)	1,2 (1,0-1,4)
Source of information consulted and trust			
Associations or religious institutions	High	1	1
	Medium	1,6 (1,3-1,8)	1,8 (1,4-2,3)
	Low	1,9 (1,5-2,3)	2,7 (2,1-3,5)
	Doesn't know/doesn't answer	1,4 (1,2-1,7)	1,7 (1,4-2,2)
Social media/internet search engines	High	1	1
	Medium	2,0 (1,6-2,6)	1,6 (1,2-2,2)
	Low	2,0 (1,5-2,6)	1,5 (1,1-2,0)
	Doesn't know/doesn't answer	1,6 (1,2-2,1)	1,4 (1,0-1,9)
Science/science online	High	1	1
	Medium	2,8 (2,6-3,0)	5,0 (4,5-5,5)
	Low	2,9 (2,3-3,7)	8,5 (6,7-10,8)
	Doesn't know/doesn't answer	1,9 (1,7-2,1)	2,8 (2,4-3,2)
Traditional mass media (*including websites*)	High	1	1
	Medium	2,1 (2,0-2,3)	3,1 (2,7-3,5)
	Low	3,5 (3,0-4,0)	11,9 (10,0-14,1)
	Doesn't know/doesn't answer	2,0 (1,7-2,4)	4,7 (3,8-5,8)
Government or institutions	High	1	1
	Medium	2,6 (2,4-2,8)	4,1 (3,7-4,6)
	Low	4,3 (3,8-4,9)	13,7 (11,8-15,9)
	Doesn't know/doesn't answer	2,0 (1,8-2,3)	4,3 (3,7-4,9)

Supplementary Table S2: Propensity to SARS-CoV-2 vaccination and vaccine hesitancy – Multivariable analysis

		Probably yes	No or probably not
		**OR (95% CI)**	**OR (95% CI)**
Sex at birth	Males	1	1
	Females	1,2 (1,1-1,3)	1,5 (1,4-1,7)
	Not pregnant	1,2 (1,1-1,3)	1,5 (1,3-1,7)
	Pregnant	1,6 (1,3-2,1)	3,3 (2,4-4,5)
Class of age	19-29	1	1
	30-39	1,2 (1,0-1,5)	1,6 (1,2-2,1)
	40-49	1,3 (1,1-1,6)	1,9 (1,4-2,5)
	50-59	1,2 (1,0-1,4)	1,6 (1,2-2,1)
	60+	0,9 (0,7-1,1)	0,8 (0,6-1,1)
Employment and work category at risk	Employed, not at risk	1	1
	Employed, school staff	1,1 (1,0-1,2)	1,0 (0,8-1,2)
	Employed, health staff	1,0 (0,8-1,2)	1,4 (1,1-1,8)
	Employed, other at risk	1,1 (1,0-1,3)	1,3 (1,1-1,6)
	Unemployed	1,1 (1,0-1,3)	1,0 (0,9-1,3)
	Students	0,9 (0,7-1,1)	0,5 (0,3-0,7)
	Retired	0,7 (0,6-0,9)	0,8 (0,6-0,9)
	Other	1,1 (1,0-1,3)	1,4 (1,2-1,7)
Educational level	High	1	1
	Medium	1,2 (1,2-1,3)	1,5 (1,4-1,7)
	Low	1,4 (1,2-1,7)	1,7 (1,4-2,1)
Deprivation score	0	1	1
	1	1,1 (1,0-1,2)	1,2 (1,1-1,3)
	2	1,2 (1,1-1,4)	1,4 (1,1-1,6)
	3+	1,3 (0,9-1,9)	1,2 (0,7-1,9)
Geographical area	Northern	1	1
	Central	1,0 (0,9-1,1)	1,0 (0,9-1,1)
	Southern and islands	0,9 (0,8-1,0)	1,0 (0,8-1,1)
	Unknown	na	na
BMI	Healthy weight	1	1
	Underweight	1,4 (1,2-1,7)	1,5 (1,2-1,9)
	Overweight	0,8 (0,8-0,9)	0,8 (0,7-0,9)
	Obesity	0,8 (0,7-0,9)	0,8 (0,6-0,9)
	Unknown	0,8 (0,6-1,0)	0,9 (0,6-1,4)
N° of comorbidities	None	1	1
	One	1,0 (0,9-1,1)	1,0 (0,9-1,1)
	Two	1,0 (0,9-1,1)	1,1 (0,9-1,3)
	Three or more	1,0 (0,9-1,2)	1,2 (1,0-1,5)
Previous COVID-19	Yes	1,2 (1,1-1,3)	1,3 (1,1-1,5)
Smoker	No	1	1
	Former smoker	0,9 (0,8-0,9)	0,9 (0,8-1,0)
	Smoker	0,8 (0,7-0,9)	0,9 (0,8-1,0)
Alcohol drinkage between meals	Never	1	1
	≤ once a week	1,0 (0,9-1,1)	0,8 (0,7-0,9)

	Two or more times a week	0,9 (0,8-1,0)	0,8 (0,7-0,9)
Perceived health status	Good	1	1
	Adequate	1,2 (1,1-1,3)	1,0 (0,9-1,2)
	Bad	1,1 (0,9-1,5)	1,5 (1,1-2,1)
Perceived stress (PSS)	Low (<14)	1	1
	Moderate (14-26)	1,0 (1,0-1,1)	0,9 (0,8-1,0)
	High (>26)	0,8 (0,6-0,9)	1,0 (0,8-1,3)
	NA	1,0 (0,8-1,2)	0,8 (0,6-1,1)
Fear of being infected with SARS-CoV-2			
for oneself	Yes, a lot	1	1
	Quite enough	1,3 (1,2-1,5)	1,2 (1,0-1,4)
	Neutral	1,5 (1,3-1,8)	1,9 (1,6-2,4)
	Just a little bit	1,7 (1,5-1,9)	2,5 (2,0-3,1)
	No, not at all	1,5 (1,3-1,8)	3,7 (3,0-4,7)
for family members	Yes, a lot	1	1
	Quite enough	1,2 (1,1-1,3)	1,2 (1,1-1,4)
	Neutral	1,4 (1,2-1,6)	1,9 (1,6-2,3)
	Just a little bit	1,4 (1,2-1,7)	2,0 (1,7-2,4)
	No, not at all	1,5 (1,2-1,9)	3,1 (2,5-3,9)
Worried about economic and working conditions			
for oneself	Yes, a lot	1	1
	Quite enough	1,0 (0,9-1,1)	0,8 (0,7-1,0)
	Neutral	1,0 (0,8-1,1)	0,7 (0,6-0,9)
	Just a little bit	0,9 (0,8-1,1)	0,8 (0,7-1,0)
	No, not at all	0,8 (0,7-1,0)	0,8 (0,6-0,9)
for family members	Yes, a lot	1	1
	Quite enough	0,9 (0,8-1,0)	1,0 (0,9-1,2)
	Neutral	1,0 (0,8-1,1)	0,9 (0,7-1,1)
	Just a little bit	0,9 (0,8-1,0)	0,8 (0,7-1,0)
	No, not at all	0,8 (0,7-0,9)	0,9 (0,8-1,1)
Perceived risk level of			
Crimes or terrorism	Low	1	1
	Medium	1,1 (1,0-1,2)	1,1 (0,9-1,2)
	High	1,2 (1,1-1,3)	1,1 (0,9-1,3)
	Doesn't know/doesn't answer	1,3 (0,8-2,0)	1,5 (0,8-2,6)
Poverty or unemployment	Low	1	1
	Medium	1,1 (0,9-1,2)	1,1 (0,9-1,3)
	High	1,1 (0,9-1,2)	1,1 (0,9-1,3)
	Doesn't know/doesn't answer	1,1 (0,7-1,7)	0,9 (0,5-1,8)
Climate change or environmental pollution	Low	1	1
	Medium	0,8 (0,7-1,0)	0,8 (0,7-1,1)
	High	0,7 (0,6-0,9)	0,7 (0,6-0,9)
	Doesn't know/doesn't answer	0,5 (0,3-0,8)	0,3 (0,2-0,7)
Contaminated food	Low	1	1
	Medium	1,4 (1,3-1,6)	1,7 (1,4-1,9)
	High	2,0 (1,8-2,3)	3,2 (2,7-3,7)
	Doesn't know/doesn't answer	1,3 (1,0-1,6)	1,6 (1,1-2,1)
Epidemics	Low	1	1

	Medium	0,9 (0,8-1,0)	0,7 (0,6-0,8)
	High	0,7 (0,6-0,8)	0,4 (0,4-0,5)
	Doesn't know/doesn't answer	1,1 (0,8-1,5)	0,7 (0,5-1,0)
Natural disasters	Low	1	1
	Medium	1,1 (1,0-1,2)	1,1 (1,0-1,2)
	High	1,2 (1,1-1,3)	1,3 (1,2-1,5)
	Doesn't know/doesn't answer	1,0 (0,8-1,4)	1,4 (1,0-1,9)
Source of information consulted and trust			
Associations or religious institutions	High	1	1
	Medium	0,9 (0,8-1,1)	0,9 (0,7-1,1)
	Low	0,9 (0,7-1,1)	0,7 (0,5-1,0)
	Doesn't know/doesn't answer	0,9 (0,8-1,1)	0,9 (0,7-1,1)
Social media/internet search engines	High	1	1
	Medium	1,3 (1,0-1,7)	1,0 (0,7-1,4)
	Low	1,3 (1,0-1,7)	0,7 (0,5-1,0)
	Doesn't know/doesn't answer	1,1 (0,8-1,4)	0,7 (0,5-1,0)
Science/science online	High	1	1
	Medium	1,9 (1,8-2,1)	2,8 (2,5-3,1)
	Low	1,5 (1,2-2,0)	2,1 (1,6-2,9)
	Doesn't know/doesn't answer	1,8 (1,6-2,1)	2,1 (1,7-2,5)
Traditional/Official mass media^	High	1	1
	Medium	1,3 (1,2-1,4)	1,6 (1,3-1,8)
	Low	1,5 (1,2-1,7)	2,9 (2,3-3,6)
	Doesn't know/doesn't answer	1,1 (0,9-1,4)	2,0 (1,5-2,6)
Government or institutions	High	1	1
	Medium	1,8 (1,6-1,9)	2,2 (1,9-2,5)
	Low	2,5 (2,2-3,0)	4,5 (3,7-5,4)
	Doesn't know/doesn't answer	1,6 (1,4-1,8)	2,5 (2,1-3,0)

^Including websites
